# Hormone Replacement Therapy and Pulmonary Hypertension: A Review of the Literature

**DOI:** 10.7759/cureus.71908

**Published:** 2024-10-20

**Authors:** Natasha K Gill, Supreet K Sohi, Girish Joseph, Neena Bhatti

**Affiliations:** 1 Internal Medicine, University Hospitals Parma Medical Center, Parma, USA; 2 Medicine, Rajendra Institute of Medical Sciences, Patiala, IND; 3 Pharmacology, Christian Medical College and Hospital, Ludhiana, IND

**Keywords:** estrogen, hormone replacement therapy, paradox, post-menopausal, pulmonary hypertension

## Abstract

Pulmonary hypertension (PH) is a multifactorial condition that encompasses a group of diseases characterized by a progressive increase in pulmonary vascular resistance and pulmonary arterial pressure, ultimately leading to right heart failure and death. The primary goals of PH treatment are to lower pulmonary arterial pressure, alleviate symptoms such as shortness of breath and chest pain, address modifiable risk factors, and manage the underlying cause, often a common advanced disease like chronic obstructive lung disease or left heart disease. While sex is an unchangeable risk factor for PH development, the presence or absence of estrogens has a significant influence on its progression. Hormone replacement therapy (HRT) is the recommended form of estrogen therapy for postmenopausal women, but only in carefully selected cases. However, a paradox arises, as some research suggests HRT benefits women, while other studies highlight its risks. This review provides a comprehensive analysis of the literature on the role of HRT in PH.

## Introduction and background

Pulmonary hypertension (PH) is a multifactorial disease characterized by a group of conditions marked by a gradual increase in pulmonary arterial pressure and pulmonary vascular resistance, eventually leading to right heart failure and death. PH is defined by a resting mean pulmonary arterial pressure exceeding 20 mmHg [1]. According to the 6th World Symposium on Pulmonary Hypertension [2], PH is classified into five categories based on pathological findings, hemodynamic features, clinical presentation, and treatment approach: pulmonary arterial hypertension (PAH), PH due to left-sided heart disease (PH-LHD), PH due to chronic lung disease (PH-CLD), chronic thromboembolic PH (CTEPH), and PH with unclear and/or multifactorial mechanisms [3].

The treatment of PH primarily addresses the underlying cause, often an advanced disease like chronic obstructive lung disease or left heart disease. In rare cases, PH may be caused by a primary vasculopathy or chronic thromboembolism. The former often requires surgical evaluation, while the latter is treated with medical therapies [4]. Prevention efforts in PH focus on reducing modifiable risk factors and improving cardiovascular health to prevent disease onset. Modifiable risk factors include lifestyle changes such as adopting a balanced diet, regular exercise, and quitting smoking, as tobacco use is a significant risk factor for PH. Additionally, managing comorbidities like chronic obstructive pulmonary disease (COPD), hypertension, and obstructive sleep apnea can delay the onset of PH [5].

Non-modifiable risk factors include age over 65, family history of PH, and genetic predisposition [6]. Genetic testing is recommended for individuals with a family history of PH to assess their risk and take preventive measures [5]. Sex is another key non-modifiable risk factor that plays a crucial role in the pathogenesis of PH. Sex differences influence not only the prevalence of PH but also its severity, treatment response, and survival outcomes [2]. In PH cohorts, the female-to-male ratio increased from 1.7:1 in the late 1980s to 4:1 in the 2000s REVEAL registry, and to 2.3:1 in younger patients (18-65 years) in the COMPERA study, reflecting a growing female predominance [2,7,8]. Despite this higher prevalence, women with PH tend to have better clinical and survival outcomes, along with more favorable hemodynamic profiles. These advantages diminish after menopause, and early menopause is a significant risk factor for PH development. Thus, the presence or absence of estrogen plays a critical role in PH progression [9].

Currently, postmenopausal women requiring estrogen are often prescribed hormone replacement therapy (HRT) [10]. However, there is a paradox: while some studies suggest that HRT is beneficial for women with PH, others indicate it may be detrimental [1].

## Review

Epidemiology and pathophysiology of PH

Ernst von Romberg documented the first case of PH in 1891, while Paul Wood was the first to describe its clinical and hemodynamic characteristics in 1952 [9]. According to a study in Asia, the estimated prevalence of PH at the population level is around 1-3%. Globally, PH affects approximately 1% of the population, with prevalence rising to 10% among individuals over 65 years of age [11]. Mortality data from the United States show that PH death rates remained steady between 1980 and 2001 but increased between 2001 and 2010, particularly among women and non-Hispanic Black individuals. From 1999 to 2019, 429,105 deaths listed PH as a contributing factor, with an age-adjusted mortality rate of 7.9 deaths per 100,000 people. The age-adjusted PH mortality rate increased by 1.9% annually, with higher rates observed in women and Black individuals in New Hampshire. The crude mortality rate for those aged 85 and older was 105.4 per 100,000. Mortality nearly doubled in patients with both left heart disease and PH compared to the general PH population [12].

Of the five PH groups, PAH, classified as Group 1 PH, is the most severe form [13]. PAH is rare, with an estimated prevalence of 15-30 cases per million population [11]. Group 2 PH, also known as PH-LHD, is the most common type, accounting for 75% of all PH cases, and includes left ventricular systolic and diastolic dysfunction as well as valvular heart disease [14]. Group 3 PH, or CLD-PH, is the second most common, affecting 25% of patients overall and up to 90% of those with severe COPD [8,11]. Group 4 PH (CTEPH) has an incidence of 26-38 cases per million adults, while Group 5 PH remains of unknown prevalence [15-18].

PH registries consistently show a higher female-to-male ratio, ranging from 1.4:1 in the UK/Ireland registry to 1.6:1 in the European COMPERA registry, and 4.1:1 in the North American REVEAL registry [7,8,19]. Although women are more likely to develop PH, men typically have poorer outcomes, with a 10% lower five-year survival rate compared to women [1].

**Figure 1:**
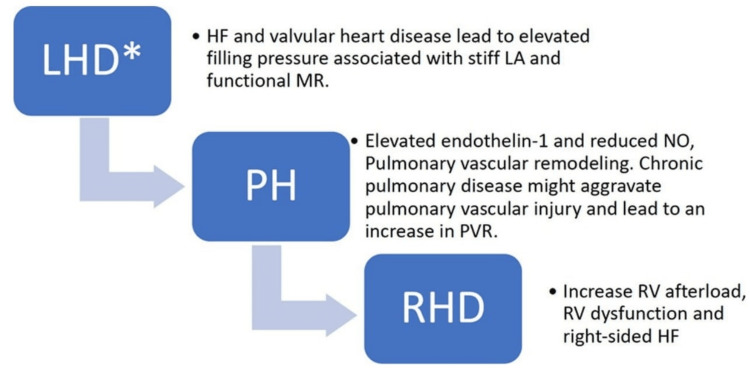
Pathophysiological progression of PH HF, heart failure; LA, left atrium; LHD, left heart disease; MR, mitral regurgitation; NO, nitric oxide; PH, pulmonary hypertension; PVR, pulmonary vascular resistance; RHD, right heart disease; RV, right ventricle Source: Al-Omary et al. (2020) [22]; reproduced with permission from the American Heart Association, Inc.

The pathogenesis of PH involves a complex interplay of multiple factors [9]. PAH, in particular, is driven by interactions between various cell types in the lung, including vascular, immune, and circulating cells. The main contributors to the disease process include pulmonary artery endothelial cells, smooth muscle cells, and fibroblasts [20,21]. A simplified illustration of the pathophysiological process is provided in Figure 1 [22].

Clinical presentation of PH

PH typically manifests as exertional dyspnea, fatigue, weakness, angina, pre-syncope, and syncope. As right ventricular (RV) failure progresses, fluid retention may occur, leading to abdominal distension and ankle edema. Physical signs can include low-volume arterial pulses, hepatomegaly, ascites, peripheral edema, a tricuspid regurgitation murmur, elevated jugular venous pressure with abnormal waveforms, an accentuated second heart sound, and an RV third heart sound. In primary care, resting ECG, ECHO, medical history, and physical examination can help assess the likelihood of PH [3].

Diagnosis and investigation

Laboratory investigations for PH include routine tests such as a complete blood count, liver function tests (LFTs), iron profile, thyroid profile, and brain natriuretic peptide (BNP) or N-terminal pro-BNP (NT-proBNP) levels. Abnormal LFT values may occur due to congestive hepatopathy from right heart failure. Since thyroid dysfunction is common in PAH and can develop at any stage of the disease, it is crucial to identify it in patients who experience sudden clinical deterioration. BNP and NT-proBNP levels are useful as independent predictors of outcomes during evaluation [5].

A normal ECG does not exclude PH; however, signs of right-sided heart strain and chamber enlargement, such as P pulmonale, right bundle branch block, right axis deviation, RV strain, and RV hypertrophy (RVH), may be present on an abnormal ECG [23]. On chest radiography, signs of underlying PH can include a water bottle-shaped cardiac silhouette, peripheral vascular pruning, pulmonary artery enlargement, and right atrial enlargement [24].

**Figure 2:**
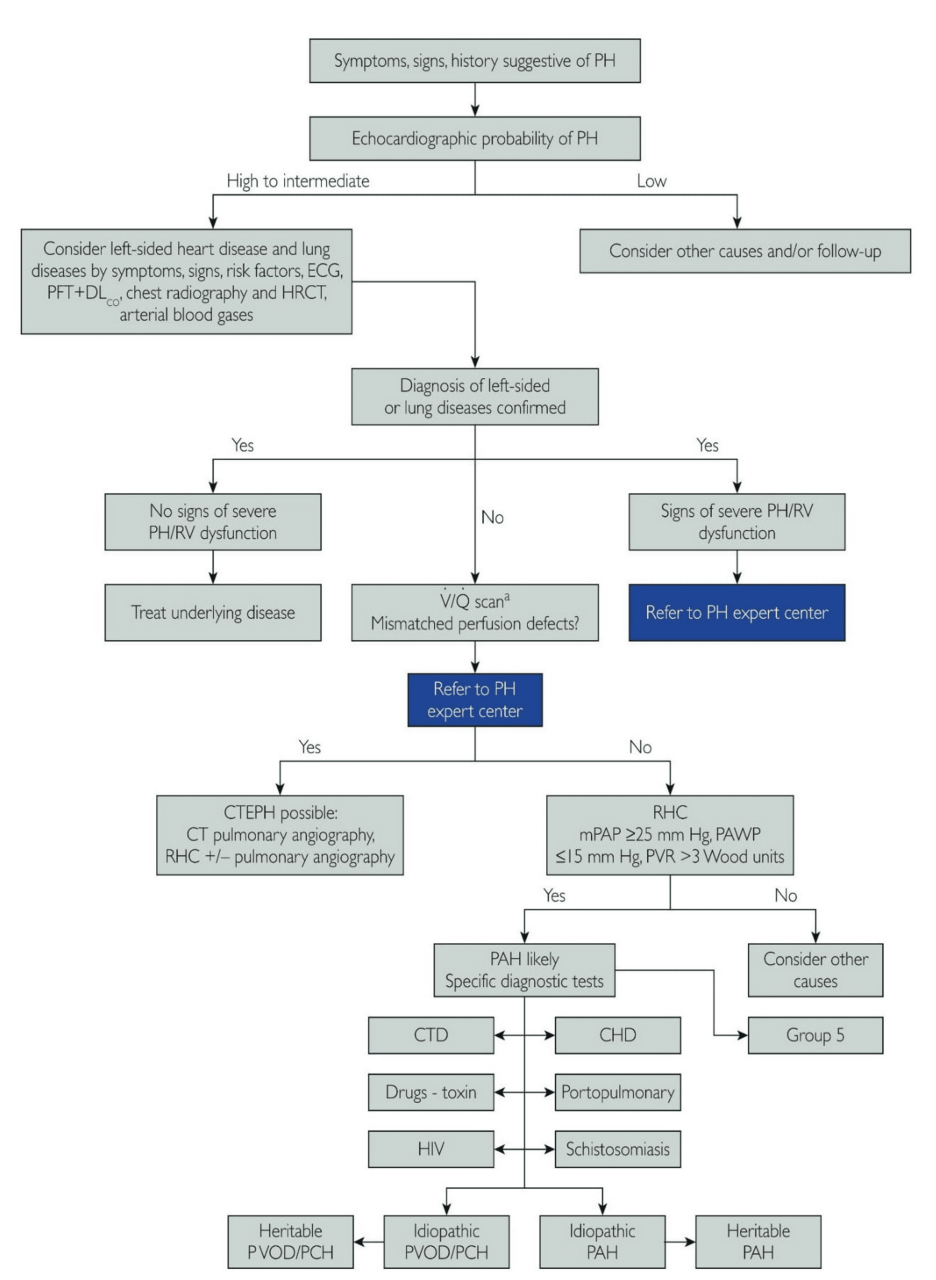
Diagnostic algorithm of PH according to the European Society of Cardiology CHD, coronary heart disease; CTD, connective tissue disease; CTEPH, chronic thromboembolic pulmonary hypertension; DLco, diffusing capacity of the lungs for carbon monoxide; HRCT, high-resolution computed tomography; mPAP, mean pulmonary arterial pressure; PAH, pulmonary artery hypertension; PAWP, pulmonary artery wedge pressure; PCH, paroxysmal cold hemoglobinuria; PFT, pulmonary function test; PH, pulmonary hypertension; PVOD, pulmonary veno-occlusive disease; PVR, pulmonary vascular resistance; RHC, right heart catheterization; RV, right ventricle; V/Q, lung ventilation perfusion scan Source: Mandras et al. (2020) [3]; reproduced with permission from Elsevier, Inc.

For PH assessment, transthoracic ECHO remains the most crucial noninvasive diagnostic tool [5]. The European Society of Cardiology/European Respiratory Society diagnostic algorithm for PH is shown in Figure 2 [3].

Standard treatment for PH

The treatment of PH varies based on the severity of the disease and the specific group to which the patient belongs [3]. The initial agents used for the treatment of PAH were prostacyclin analogues, which are potent vasodilators that target the prostacyclin receptor, and phosphodiesterase inhibitors (PDEIs) [25]. Prostacyclin analogues include intravenous epoprostenol, oral beraprost, intravenous and subcutaneous treprostinil, and iloprost. Endothelin receptor antagonists, such as bosentan, macitentan, and ambrisentan, act as vasodilators by blocking the vasoconstrictive effects of endothelin I [3]. PDEIs like sildenafil and tadalafil induce vasodilation by increasing levels of cyclic adenosine monophosphate [9]. For patients with PAH receiving pharmacological therapy, psychosocial support and a supervised exercise training program are recommended [5].

For patients with type 2 PH, also known as PH-LHD, the primary focus remains on managing the underlying heart and lung conditions. Generally, PH-LHD is associated with a poor prognosis, and nonspecific vasodilators are utilized for management [25]. Lung transplantation is currently the only potentially curative option for patients with PH-CLD, the third type of PH. No proven medical treatments exist for PH-CLD, and there is no evidence that medications approved for PH are beneficial for this group [26]. For patients with CTEPH, pulmonary thromboendarterectomy is the preferred treatment option, as it can provide a cure, significant symptom relief, and improvements in hemodynamics and RV function in most cases [25,27]. Riociguat is the only approved medical therapy for CTEPH, indicated for adults with inoperable or persistent/recurrent disease [26]. For group 5 PH, managing the underlying condition is essential, and patients require continuous monitoring, symptomatic treatment, and ongoing evaluation of disease progression and response to therapy [5].

Recent advances in PH management include inhaled treprostinil, anticoagulants, recombinant fusion proteins, and stem cell therapy [28]. Additionally, emerging evidence suggests that sex steroids, such as estrogen and progesterone, may positively influence lung function in women [10]. HRT, which combines estrogen and progesterone, mimics the hormones produced by the ovaries in humans [29].

HRT and its role in PH

HRT is a treatment modality used for women during the menopausal transition [29]. It is highly effective for managing osteoporosis, vaginitis, vasomotor symptoms, and other postmenopausal issues. For women without a uterus, HRT consists of estrogen alone; for those with a uterus, it is administered as a combination of estrogen and progesterone [10]. Estrogen, primarily secreted by the ovaries in premenopausal women, is essential for reproductive function and the development and maintenance of secondary sexual characteristics. In contrast, progesterone is secreted by the adrenal cortex, ovaries, and, in small amounts, by the testes. Its primary functions include regulating the menstrual cycle, maintaining pregnancy, and supporting reproductive processes [1].

The hypothalamic-pituitary-gonadal (HPG) axis plays a crucial role in sexual development. The hypothalamus releases gonadotropin-releasing hormone, stimulating the pituitary gland to secrete luteinizing hormone and follicle-stimulating hormone. These hormones regulate the production of other sex hormones in the gonads, such as testosterone, progesterone, and estrogen [10]. It is important to note that alterations in the HPG axis can significantly impact reproductive health [30]. Following menopause, the production of sex hormones declines sharply, resulting in the loss of hypothalamic feedback inhibition. This disruption in the delicate balance of the HPG axis during menopause is due to the ovaries’ decreased synthesis of progesterone and estrogen, leading to changes in hormonal control and reproductive function [31].

**Figure 3:**
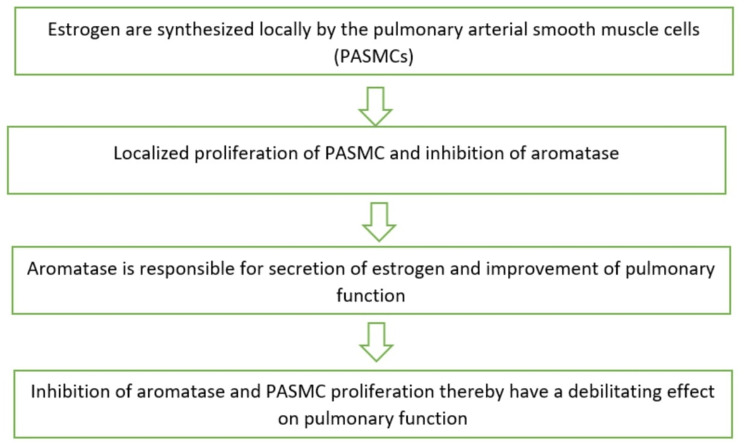
Estrogen depicting female bias Source: Hye et al. (2021) [32]; reproduced with permission from the American Physiological Society

While females are more susceptible to developing PH, they also exhibit a better response to treatment and higher survival rates compared to men [2]. These sex differences have been termed the “estrogen paradox,” which can be explained as follows: evidence supporting the notion that the female bias is influenced by estrogen [32], as illustrated in Figure 3.

On the other hand, evidence supporting the notion that estrogen contributes to the higher survival rates observed in females includes the following findings: Estrogen enhances RV function and offers protection against RVH. In animal models, pretreatment with estrogen has been shown to slow the progression of PH. Furthermore, estrogen exhibits anti-proliferative effects on pulmonary arterial endothelial cells. Additionally, animals that have undergone surgical removal of their ovaries develop a more severe form of PH [32].

Clinical outcomes of HRT

Research has demonstrated that estrogen plays a protective role in the vasculature by reducing inflammatory and atherosclerotic processes [33]. Moreover, estrogen functions as a hypotensive agent and vasodilator, promoting vascular relaxation by acting directly on vascular smooth muscle or by stimulating endothelium-derived vasodilatory molecules such as nitric oxide. The cardiovascular effects of estrogen are associated with benefits in both the quality and quantity of life. However, it is crucial to recognize that estrogen can also have detrimental effects on other tissues [10]. This dual nature of estrogen is referred to as a dimorphic response, as its impact on RV function is a key prognostic factor in PH [2].

A theory known as the three-tier concept provides an explanation for the contradictory effects of estrogen observed in experimental animal models and patients with PAH. According to this theory, estrogens may act as instigators of vascular injury in the pulmonary circulation, leading to the development of PH. Conversely, estrogens may also serve as protectors of RV function by inducing vasodilation of the RV wall, contributing to greater survival rates in women compared to men [34]. Furthermore, the effects of estrogen on the cardiovascular system can vary over time. Observations indicate that HRT administered more than 10 years after the onset of menopause no longer offers advantages. This complexity necessitates careful monitoring and individualized treatment dosages to address the unique needs of each patient when selecting the appropriate estrogen therapy [35].

In a recent clinical trial funded by the National Heart, Lung, and Blood Institute, the Pulmonary Vascular Disease Phenomics study posited that HRT may improve outcomes in PH. The study considered both endogenous and exogenous estrogen exposure. Endogenous hormones are those produced by women’s bodies before menopause, while exogenous hormones are those administered through HRT. Exogenous hormone exposure was assessed based on self-reported HRT use, while endogenous exposure was evaluated based on the length of reported menstrual cycles. Conducted with over 700 women, the study demonstrated that average pulmonary arterial pressure decreased with longer lifetime menstrual cycles across all PH groups. Additionally, HRT use was associated with higher RV fractional shortening and lower mean pulmonary artery pressure [36].

However, a large, single-center case-control study conducted in 2020 found no significant differences in endogenous or exogenous sex hormone exposure regarding the development of PH [37]. It is essential to acknowledge that sex differences are present across all groups of PH. In Group 1 PH, the estrogen paradox indicates that while women have a higher risk of developing the disease, once affected, they demonstrate a better response to treatment and longer survival compared to men [2]. This paradox has a genetic basis, as mutations in the bone morphogenetic protein receptor 2 (BMPR2) play a contributory role in patients with PAH. Estrogen has been shown to suppress the BMPR2 gene, leading to decreased BMPR2 expression, which promotes PAH [38]. Conversely, improvements in RV ejection fraction are greater in females than in males, potentially offsetting the negative impact of estrogen in women [39]. Despite the higher prevalence of other forms of PH, statistically significant effects of estrogen have not been observed in these groups [2,36].

## Conclusions

PH is a condition that occurs more frequently in females than in males, yet women tend to exhibit better outcomes and survival rates. This disparity highlights the critical role that sex hormones play in the pathogenesis of PH. HRT, which primarily consists of estrogen, has been extensively studied for its potential impact on women with this condition. Research findings regarding the use of HRT in PH have been conflicting. Some studies emphasize the detrimental effects of estrogen in HRT, suggesting that it may contribute to the progression of PH, while others highlight its beneficial role in promoting RV dilation and improving lung vasculature. This review aims to evaluate whether women with PH can benefit from HRT as a therapeutic option. While preliminary studies have shown promising data indicating that HRT may improve outcomes in women with PH, there is currently a lack of placebo-controlled or randomized controlled trials comparing standard therapies with HRT. Consequently, no consensus has been established regarding the use of HRT in this context. Therefore, further research is necessary to elucidate the potential clinical outcomes of HRT in patients with PH.
